# Perspective of Cyclin-dependent kinase 9 (CDK9) as a Drug Target

**DOI:** 10.2174/138161212800672750

**Published:** 2012-07

**Authors:** Vladimír Kryštof, Sonja Baumli, Robert Fürst

**Affiliations:** 1 Laboratory of Growth Regulators, Faculty of Science, Palacký University & Institute of Experimental Botany ASCR, Šlechtitelů 11, Olomouc, 783 71, Czech Republic; 2 Northern Institute for Cancer Research, University of Newcastle, Framlington Place, Newcastle upon Tyne, NE2 4HH, UK; 3 Department of Pharmacy, Center for Drug Research, Pharmaceutical Biology, University of Munich, Butenandtstr. 5-13, 81377 Munich, Germany

**Keywords:** Cancer, inflammation, kinase, P-TEFb, inhibitor, therapeutics, angiogenesis.

## Abstract

Deregulation of cyclin-dependent kinases (CDKs) has been associated with many cancer types and has evoked an interest in chemical inhibitors with possible therapeutic benefit. While most known inhibitors display broad selectivity towards multiple CDKs, recent work highlights CDK9 as the critical target responsible for the anticancer activity of clinically evaluated drugs. In this review, we discuss recent findings provided by structural biologists that may allow further development of highly specific inhibitors of CDK9 towards applications in cancer therapy. We also highlight the role of CDK9 in inflammatory processes and diseases.

## INTRODUCTION

The cyclin-dependent kinase 9 (CDK9) is a serine/threonine kinase that forms the catalytic core of the positive transcription elongation factor b (P-TEFb) [1-4]. This enzyme is critical for stimulating transcription elongation of most protein coding genes, including key developmental and stimulus-responsive genes, by RNA polymerase II (RNAPII) [5]. RNAPII is paused soon after transcription initiation by DSIF and NELF [6]. CDK9 is then recruited to the paused transcription complex where it phosphorylates DSIF and NELF as well as the RNAPII C-terminal domain, and thereby releases RNAPII from the pause site. Activity of CDK9 is dependent on binding to a regulatory cyclin subunit (cyclin T1, T2a or T2b) and is further regulated through association with other macromolecules. These modulators include activators like c-myc [7], NF-κB [8], androgen receptor (AR) [9], Brd4 [10;11], or subunits of the super elongation complex [12-14] and inhibitory proteins or complexes such as the inhibitory 7SK small nuclear RNA (7SK snRNA) containing complex [15-17].

CDK9 belongs to a family of 13 protein kinases that share sequence homology and dependence upon the binding of a cyclin subunit for activation. Other members of this family are also transcriptional regulators. CDK7/cyclin H is a component of the general transcription factor TFIIH and is involved in transcription initiation [18], CDK7 is also known as the CDK-activating kinase (CAK), which activates other CDKs by phosphorylation of the activation segment. CDK8/cyclin C and CDK11/cyclin L are involved in mRNA splicing [1]. CDKs 12 and 13 (both activated by cyclin K) regulate, like CDK9, transcription elongation by phosphorylating the RNAPII CTD [19,20]. Facilitated by their localization close to chromatin, CDKs 12 and 13 play additional roles in maintenance of genome integrity [2,20]. 

Besides transcription, CDKs regulate other cellular processes and were originally identified as the core of the cell cycle control system [3,21]. For example, CDKs 4 and 6, complexed with cyclin D, link mitogenic signalling to initiation of G1 phase-specific transcription by phosphorylating the Rb protein, while CDK2/cyclin A governs DNA replication during S phase, and CDK1/cyclin B is the master regulator of mitosis, orchestrating and effecting chromosome condensation and nuclear envelope break-down. The activity of CDKs during the cell cycle is primarily regulated by the balance between expression and degradation of cyclins, which are usually present in the cell in a phase-specific manner. Furthermore, enzymatic activity of CDKs is modulated by their phosphorylations (both positive and negative), intracellular localization and by the presence of endogenous protein inhibitors [3;21], apparent especially at the checkpoints, where progression may be blocked until stage-specific processes have completed satisfactorily.

The complicated system controling progression through the cell cycle allows cells to coordinate duplication of chromosomes and their equal distribution between both daughter cells with enormous precision. Any disturbances to the system may eventually lead to qualitative and quantitative changes of DNA content in the nucleus, which is often a reason for serious diseases including cancers. Accordingly, CDKs and especially their modulators and substrates have frequently been found to be deregulated in cancer cells. These aberrations in CDK activities led therefore to an intensive search for CDK inhibitors for therapeutic applications [22,23].

**Fig. (1):**
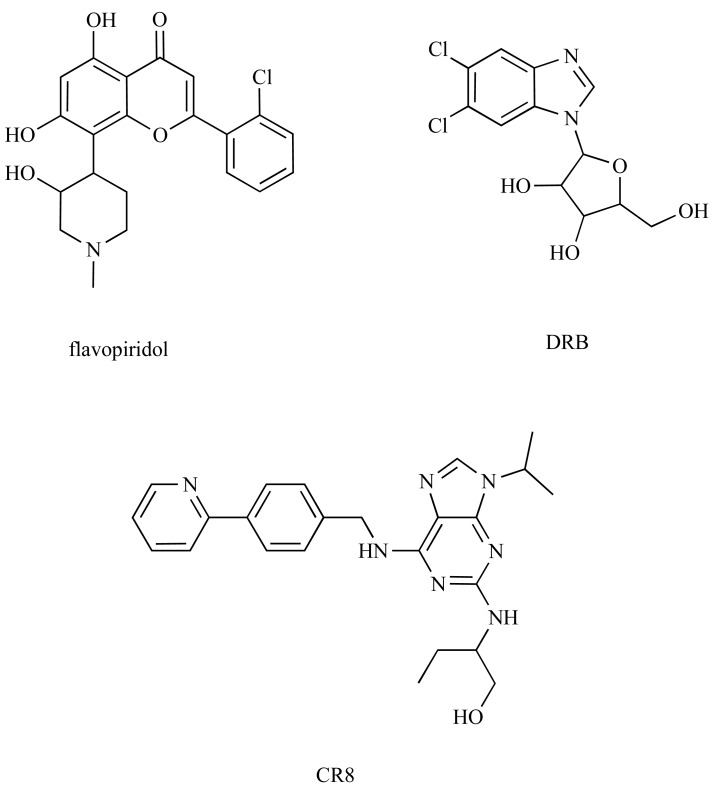
CDK inhibitors for which cocrystal structures with CDK9/cyclinT are available.

Flavopiridol and roscovitine are the most intensively studied CDK inhibitors tested in anticancer clinical trials. Both compounds are pan-selective CDK inhibitors; roscovitine inhibits equipotently CDK2, 5, 7 and 9, flavopiridol has a preference for inhibition of CDK9 over other CDKs (Ki <3 nmol/L compared with Ki values of 40 to 70 nmol/L for cell-cycle CDKs [24]). While both inhibitors influence cell cycle regulation through inhibition of CDKs 1, 2 and 4, recent work suggests that targeting CDK9 might be critical for their anticancer activity [25-29]. In addition, it has been suggested that deregulation of CDK9 activity and CDK9-interacting proteins are associated with several other human diseases, including acquired immunodeficiency syndrome (AIDS) or cardiac hypertrophy [4,30]. Therefore CDK9 emerges as a possible therapeutic target [31]. Detailed biochemical characterization of CDK9, as well as the availability of crystal structures of its complexes with cyclin T, HIV TAT protein, physiological substrate ATP and several small molecule inhibitors (Fig. **1**) can now form the basis for rational drug development. 

## CDK9 STRUCTURE AND IMPLICATIONS FOR STRUCTURE-BASED INHIBITOR DESIGN 

**Fig. (2):**
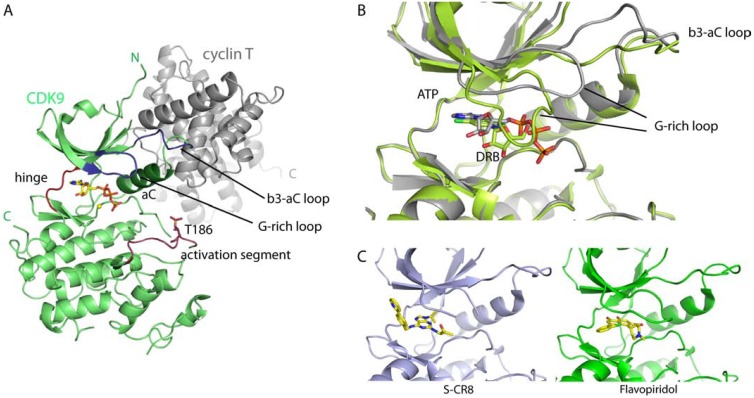
Structure and inhibitor binding of CDK9. A: Ribbon model of CDK9/cyclin T (pdb id: 3BLQ) in green and grey, respectively. ATP is represented as a yellow stick model and the different elements of the kinase fold are indicated. B: Superposition of CDK9/cyclin T ATP (grey, pdb id: 3BLQ [33]) and DRB (lime green, pdb id: 3MY1 [37]) bound structures. Both structures where superposed on their C-terminal kinase domains. CDK9 is represented as ribbon, ATP and DRB as stick models, respectively. C: Structures of CDK9 bound SCR8 (pdb id: 3LQ5 [36]) and flavopiridol (pdb id: 3BLR). Same view as in B.

CDK9 has a typical protein kinase fold consisting of an N- and a C- terminal kinase lobe and a short C-terminal extension (Fig. **2A**). The ATP-binding site is located between the N- and C-terminal lobes and contains highly conserved active site residues that coordinate ATP binding and phosphotransfer. Like other CDKs, CDK9 is activated by the association with a cyclin, cyclin T, and phosphorylation of a threonine residue in the activation segment, T186. By analogy to CDK2, phosphorylation leads to the formation of a stable structure that recognizes the proline residues at the +1 position of CDK9 substrates and orients them for phosphorylation [32,33]. In contrast to the large interaction interface of CDK2/cyclin A, the CDK9/cyclin T interaction is limited. The N-terminal domain of cyclinT interacts only with the N-terminal kinase lobe to support the αC-helix in its active conformation (Fig. **2A**). The smaller interaction interface leads to fewer restraints on the orientation of the CDK9 N- and C-terminal kinase domains. It has been suggested that regulatory factors may bridge between the C-terminal kinase and cyclin domains to regulate kinase activity and HIV-TAT has subsequently been shown to exploit this binding mode [33,34]. 

High sequence conservation among kinases and especially members of the CDK family, make it challenging to generate selective CDK9 inhibitors [30;31]. However, the structural comparison of catalytic kinase domains gives insights into small differences of the architecture of the ATP binding site and is a powerful tool to use in developing inhibitors that are specific for a certain kinase [35]. Co-crystal structures of known CDK9 inhibitors like flavopiridol, CR8 and 5,6-dichlorobenzimidazone-1-β-D-ribofurano-side (DRB) with CDK9 and CDK2 allowed the comparison of the inhibitor binding modes to the two kinases [33;36;37]. While flavopiridol and CR8 bind in the same way in the ATP site of both enzymes, DRB adopts a kinase specific orientation. The two chlorine atoms of DRB form halogen bonds to the CDK9 hinge region and exploit small geometrical differences to orient the inhibitor [37], suggesting that chlorine-halogen bond formation might be used to target inhibitors to the CDK9 hinge region with some specificity (Fig. **2B**).

Kinases can adopt a range of different conformational states that are often characteristic for a specific enzyme and can offer further possibilities for targeted inhibitor design. Two different crystal systems for the active CDK9/cyclin T and CDK9/cyclin T/TAT complexes have been reported [33,34]. The two systems trap different conformational states of the catalytic kinase subunit, reveal variability in the relative dispositions of the N- and C-terminal kinase lobes, and identify the glycine-rich loop, the β3-αC loop and the αC helix as flexible elements.

It appears that inhibitors showing specificity for CDK9 over other CDKs exploit this conformational flexibility. Co-crystal structures of CDK9/cyclin T with flavopiridol and DRB show a downward movement of the glycine-rich loop to optimally accommodate the respective inhibitors [33,37]. This conformation is further stabilized by a concomitant relocation of the β3-αC loop, which now overlaps with the original position of the glycine-rich loop (Fig. **2B**, **C**). Deschloroflavopiridol and DRB binding do not lead to the same conformational changes in CDK2/cyclin A, indicating that this particular arrangement is CDK9 specific [37,38]. In contrast the pan-CDK inhibitor CR8 induces a downward movement of the glycine-rich loop in both CDK9 and CDK2 [36]. However, the CDK2 ATP-binding site differs from that of CDK9 in that the conformational flexibility of the CDK2 β3-αC loop is independent of the position of the glycine-rich loop [39]. 

By compositing the family of inhibitor-bound CDK9 structures that adopt a glycine-rich loop down conformation, it is possible to define the space in the ATP-binding site that can be exploited in the design and further optimization of high affinity CDK9 inhibitors (Fig. **2B**). An additional factor that may influence the shape of the CDK9 ATP-binding site is its little-conserved C-terminal sequence, which has to be taken into account when testing inhibitors for their affinity and specificity. This extension of the kinase domain was shown to increase inhibition of CDK9 by DRB and stabilize the protein inhibitor complex [37]. It also changes the binding mode of EXEL-8647 to CDK9 [30]. Enzymatic assays and differential fluorescence scanning experiments [40] have been used to assess the C-terminal dependence of inhibitor binding. 

## THE ROLE OF CDK9 IN THE ANTICANCER ACTIVITY OF CDK INHIBITORS

To date, over 20 potent CDK inhibitors undergo phase I-II clinical trials in patients with different cancers [22,23,41]. The consequence of administering CDK inhibitors was originally expected to be restoration of normal cell cycle in transformed cells that proliferate rapidly and with compromised check points. Indeed all CDK inhibitors that target cell cycle kinases, such as for example flavopiridol, roscovitine or AT7519, display cytostatic activity mediated by inhibition of CDK1, 2 and 4 [42;43]. However, the antitumour activities of these inhibitors are both highly complex and pleiotropic. It took some time to understand why application of CDK inhibitors, once believed to target only cell cycle CDKs, leads to the induction of cell death. Clear explanation was published by Shapiro *et al.*, who demonstrated that depletion of CDKs 1 and 2 has cytostatic effects, while simultaneous inactivation of CDKs 1, 2 and 9 induces apoptosis in cancer cell lines [25]. 

Today it is widely accepted that the diverse effects of CDK inhibitors is partly due to their ability to block multiple CDKs involved in both cell cycle and transcription regulation. The role of CDK9 in regulating transcription has been identified as the target of most CDK inhibitors that have entered clinical trials. CDK9 inhibition leads to the downregulation of transcriptionally inducible genes with short half-lives, including cell cycle regulators and antiapoptotic factors [44]. Although the drugs target several CDKs, it has been proposed that the induction of apoptosis arises primarily due to the inhibition of CDK9 [26-44]. Inhibition of transcrip-tion (brought about by inhibiting CDK9) leads to a rapid decrease not only of D-type cyclins, that support uncontrolled proliferation, but also of antiapoptotic proteins XIAP and Mcl-1. Similarly, the induction of chronic lymphocytic leukemia cell death induced by flavopiridol, SNS-032 or roscovitine is also mediated by the repression of transcription due to CDK9 inhibition and concomitant down-regulation of XIAP and Mcl-1 [45-47]. A dependence on the expression of antiapoptotic proteins is not exclusive to multiple myeloma or chronic lymphocytic leukemia. Experimental compound CR8, a congener of roscovitine, potently induces apoptosis in neuroblastoma cells, accompanied by Mcl-1 down-regulation both at the mRNA and protein levels [48]. Similarly, compound VER-54505 reduced levels of Mcl-1 and XIAP in human osteosarcoma cell line U2OS [49].

Apart from its antiproliferative and cytotoxic activites caused by inhibition of cell-cycle and transcriptional CDKs, flavopiridol significantly potentiates the effects of topoisomerase I poisons by suppressing Rad51 expression [50]. Rad51 is a protein involved in homologous recombination and DNA repair and its down-regulation after flavopiridol treatment results in enhancement of apoptosis. This could be the rationale for the observed clinical activity of flavopiridol in combination with DNA damaging drugs [51].

CDK9 is often aberrantly activated by oncogenic fusion proteins triggering distinctive lymphoid and myeloid leukemias. In particular, the histone methyltransferase MLL forms various fusions with regulators of chromatin modification and transcriptional elongation, including proteins of the CDK9 containing SEC [12,13,52]. In this light, it is interesting to note that at least two CDK inhibitors, including the newly described macrocyclic compound TG02, blocked proliferation and induced apoptosis of acute myeloid leukemia cells with MLL rearrangements both *in vitro* and *in vivo* [53,54].

As a part of the positive transcriptional regulatory complex, CDK9 interacts also with the androgen receptor (AR) to enhance transcription activity [9]. CDK9 regulates the androgen receptor through S81 phosphorylation and this is an important step in regulating not only its transcriptional activity, but also for prostate cancer cell growth [55]. It has been shown that pharmacological inhibition of CDK9 by flavopiridol resulted in decreased AR transcription and proliferation rates, which are further potentiated by AR antagonist [55]. 

Unexpectedly, some CDK inhibitors (including flavopiridol, SNS-032 and roscovitine) have also been shown to inhibit angiogenesis *in vitro* [56-60]. Although these inhibitors demonstrate different kinase-selectivity profiles, so that their respective mechanisms of inhibiting angiogenesis may differ, they all share significant activity against CDK9. The mechanism responsible for the anti-angiogenic properties of both flavopiridol and SNS-032 have therefore been partially ascribed to down-regulation of both mRNA and protein levels of VEGF, the most potent tumour angiogenic factor [56;57]. A connection between angiogenesis, mRNA transcription and CDK9 has been further suggested by analyses of the effects of 4-amino-6-hydrazino-7-β-D-ribofura-nosyl-7*H*-pyrrolo-pyrimidine-5-carboxamide (ARC) and 4-(4-hydroxyphenyl)azo-3,5-diamino-1*H*-pyrazole (CAN508). Both these compounds were originally identified as inhibitors of transcription, but they also have anti-angiogenic activity *in vitro* [58,59,61,62]. Anti-angiogenic potential of CDK9 inhibitors has been highlighted by the finding that a mutation of HEXIM1, a negative regulator of CDK9 activity, leads to increased VEGF and HIF-1α expression in murine mammary glands [63]. However, we recently found that CDK5 also plays an important role in angiogenesis. The anti-angiogenic activity of several CDK inhibitors with different structures, including roscovitine, arises at least partially from interference with CDK5 [60,64].

## THE INVOLVEMENT OF CDK9 IN INFLAMMATORY PROCESSES

### The Role of CDK9 in Inflammatory *In vivo* Models

The precise role of CDK9 in inflammatory processes *in vivo* would best be assessed in CDK9-deficient mice. Unfortunately, there are no reports available about attempts to generate these mice due to low chances to obtain viable animals: Kohoutek *et al. *[65] showed that cyclin T2, which together with CDK9 forms P-TEFb, is essential in mouse embryogenesis. The deletion of cyclin T2 did not even allow the appearance of cyclin T2^-/-^ embryos, not to mention pups or adult mice. In *C. elegans*, the knock-down of CDK9 led to embryonic lethality [66] and CDK9-silenced fruit flies died during metamorphosis [67]. Consequently, because there are no genetically altered mice at hand, the current research depends on the usage of kinase inhibitors. Flavopiridol was reported to inhibit inflammatory processes in two different mouse models:

- Flavopiridol is able to dose-dependently suppress murine collagen-induced arthritis, even when applied therapeutically, that is after the clear clinical manifestation of joint swelling [68]. Hallmarks of arthritis, such as cartilage and bone damage or leukocyte-infiltrated pannus tissue, were nicely diminished in flavopiridol-treated mice. In this setting, flavopiridol did not interfere with lymphocyte functions, whereas it strongly reduced the proliferation of synovial fibroblasts. This growth inhibition did not lead to an induction of apoptosis. Interestingly, even in lymphocyte-deficient mice flavopiridol was able to mitigate arthritis, confirming that the compound indeed blocks arthritis without affecting lymphocyte function. Regarding the molecular target(s) of flavopiridol, the authors suggested that the inhibition of CDK4 and 6 plays a crucial role in their model, since a CDK4/6-selective inhibitor showed comparable pharmacological actions. Perhaps surprisingly, the authors did not describe CDK9 as another potential target of flavopiridol in arthritis.
- We have recently proved that flavopiridol effectively limits murine hepatitis induced by concanavalin A (ConA), which triggers a strong and rapid leukocyte-dependent inflammatory liver injury [69]. The compound inhibited the ConA-evoked rise in serum transaminases and protected against neutrophil infiltration of the liver tissue. Interestingly, as observed also in the above described arthritis model, leukocyte function was not altered. However, flavopiridol strongly decreased the interaction of neutrophils with endothelial cells (ECs) by blocking adhesion molecule expression in ECs. Most impor-tantly, we could identify CDK9 as the primary target of flavopiridol responsible for its profound anti-inflammatory effects *in vitro*. In accordance with this finding, we could also demonstrate that the anti-inflammatory action of roscovitine derives partly from its inhibition of both CDK5 and CDK9 [70].

### CDK9 and Leukocytes

In 1998, three publications suggested an association of CDK9 with the differentiation and function of normal healthy (non-tumoral) lymphocytes: (i) Arguello *et al. *[71] reported that flavopiridol diminishes the number of lymphocytes in different immune organs of normal healthy mice, such as the spleen or thymus, by inducing apoptosis. The mitogen-induced proliferation of isolated human lymphocytes was also shown to be decreased. (ii) Herrmann *et al.* [72] then showed that CDK9 mRNA and protein levels strongly increase upon PHA- or PMA-triggered activation of quiescent human peripheral blood lymphocytes (PBLs) and CD4+ T cells. (iii) Finally, this was confirmed by Garriga *et al.* [73], who also showed that the expression of CDK9 is upregulated upon stimulation of human PBLs by PHA, PMA, or TNFα. In parallel, cyclin T1 expression is also augmented. Consequently, the increased protein concentrations lead to an increase in kinase activity of the CDK9/cyclin T1 complex. Later studies confirmed and expanded these basic findings [74-76]. CDK9 protein levels were found to change during differentiation and activation of B lymphocytes: In memory and in activated human B cells the expression of CDK9 is increased in comparison to naïve and quiescent cells, respectively [77]. Taken together, flavopiridol can induce lymphocyte apoptosis, and CDK9 is associated with the proliferation and differentiation of lymphocytes. Thus, one could hypothesize that inhibition of CDK9 might precipitate immuno-suppressive actions, thereby leading to beneficial effects parti-cularly in lymphocyte-driven inflammatory disorders. However, as mentioned above, lymphocyte function was not affected in flavopiridol-treated arthritis mice, which might argue against this hypothesis. Further *in vivo* pharmacological investigations are needed to clarify the potential of CDK9 inhibition in this regard.

In contrast to lymphocytes, CDK9 levels are not altered during the macrophage differentiation processes [78]. However, a very interesting role of CDK9 has been described in primary human macrophages [79], the anti-inflammatory cytokine IL-10 inhibits transcription of the TNF gene, coding for TNFα, by influencing transcription elongation in a gene-specific manner: IL-10 blocks the p65-mediated recruitment of CDK9 to the TNF gene, but not to the NFκBIA (coding for IκBα) promoter. Thus, the “modulation of transcription elongation by CDK9” has been highlighted as a “unique negative regulatory checkpoint within the human innate immune system” [79]. Regarding a putative role for CDK9 in the activation of macrophages, Haque *et al.* [80] recently demonstrated that flavopiridol reduces the production of TNFα and NO as well as the activation of NF-κB, IKK, p38 MAPK, JNK, and ERK in LPS-activated RAW cells (mouse leukemic/monocyte macrophage cell line). This suggests an anti-inflammatory potential of flavopiridol in the context of LPS-associated immune responses. Although not in leukocytes, an influence of flavopiridol on the activation of these dominant pro-inflammatory signal transducers has been confirmed by Takada *et al.* [81], who demonstrated that flavopiridol inhibits the activation of JNK/AP-1, p38 MAPK, JNK, and ERK, as well as the expression of ICAM-1 upon TNFα treatment in different cancer cell lines. Surprisingly, in the latter two publications, the authors did not discuss any role of CDK9 in the processes they investigated. However, the results indicate that, not only NF-κB, but also the MAPK cascade can in principle be influenced by flavopiridol, which might contribute to the overall anti-inflammatory action of this substance *in vitro* and *in vivo*.

### CDK9 and the Pro-inflammatory Transcription Factors NF-κB and STAT3

The transcription factor NF-κB plays a prominent role in inflammation, since it is involved in the expression of a wide variety of inflammatory gene products, such as cytokines or adhesion molecules. Indeed, NF-κB (p65) binds to and requires P-TEFb for transcription elongation [8]. By using DRB and a kinase-deficient CDK9 mutant, P-TEFb/CDK9 kinase activity was shown to be necessary for transcription by NF-κB. Interestingly, CDK9 (and cyclin T1) were immunoprecipitated from the TNFα-activated promoter of IL-8, whose transcription is strongly NF-κB-dependent. In 2008, Nowak *et al.* published a study [82], in which they provide evidence that the Ser276 phosphorylation of p65 is important for the activation of specific NF-κB-dependent genes, such as IL-8 and GROβ, but not IκBα, and that the association of p65 with CDK9/cyclin T1 is critical in this process. Consequently, both flavopiridol and the knockdown of CDK9 by siRNA were revealed to inhibit the expression of IL-8 and GROβ, but not of IκBα. This study implies that the pharmacological inhibition of CDK9 might be a valuable anti-inflammatory strategy. In fact, we could show that the inhibition of NF-κB via blocking CDK9 leads to strong anti-inflammatory effects in human endothelial cells [69]: both flavopiridol and gene silencing of CDK9 led to a greatly decreased ICAM-1 expression, which is responsible for leukocyte recruitment into inflammed tissues. We found that flavopiridol did not interfere with the TNFα-triggered activation cascade of NF-κB, comprising events such as IKK activation, phosphorylation of IκBα or p65, nuclear transloction of p65, and NF-κB binding to DNA. In contrast, Takada and Aggarwal reported that each of these events is nicely blocked by flavopiridol in different leukemia cell lines [83]. This discrepancy has not been investigated in detail yet, but a simple explanation would be that flavopiridol exhibits strong cell type-dependent actions. Takada and Aggarwal also showed that flavopiridol can block the expression of COX-2 and MMP-9, two typical pro-inflammatory enzymes. The involvement of CDK9 in NF-κB-dependent gene transcription was also analyzed by Huang *et al.* [84]: They found that Brd4, a bromodomain-containing protein, binds to acetylated p65 and thereby coactivates NF-κB in cooperation with p300. Brd4, in turn, binds to P-TEFb and activates CDK9. By using DRB as well as CDK9 siRNA, the activity and presence of CDK9 was shown to be required for Brd4 to coactivate TNFα-evoked NF-κB activity. Taken together, there is good evidence that CDK9 is an important regulator of specific NF-κB-driven pro-inflammatory genes and that the anti-inflammatory properties of flavopiridol are, at least in part, a result of a blockade of the CDK9-activated NF-κB pathway.

STAT3 represents another very important pro-inflammatory transcription factor; CDK9 binds to STAT3 during the IL-6-induced upregulation of the gene p21^waf1^. Accordingly DRB is able to suppress the transcription of the p21 gene [85]. The association of CDK9 and STAT3 was also confirmed by Hou *et al.* [86], who demonstrated that IL-6 induces the formation of a nuclear STAT3/CDK9 complex during hepatic acute phase response. This interaction is mediated via the STAT3 NH2-terminal domain. By using either flavopiridol or a kinase deficient CDK9 mutant or a CDK9 gene silencing approach, the crucial role of CDK9 for the IL-6-triggered upregulation of γ-fibrinogen (a typical acute phase gene product) was clearly proven.

Taken together, these data indicate that the inhibition of CDK9 represents an interesting strategy to impede the activation of the two prominent pro-inflammatory transcription factors NF-κB and STAT3. The inhibition of NF-κB has been shown to be an important mechanism by which flavopiridol exerts its anti-inflammatory actions [69;70]. Data about STAT3 are lacking in this regard.

### CDK9 Binds to TRAF2 and gp130

In 1998, MacLachlan *et al.* [87] performed a yeast two-hybrid screening of a mouse embryonic library to identify novel CDK9-binding proteins in the context of mouse developmental processes. Interestingly, they disclosed TRAF2, an important protein of the TNF receptor complex, to bind to CDK9 in the C-terminal region of the TRAF2 protein. Moreover, they showed that CDK9 is present both in the nucleus and in the cytosol. By transfection of a kinase-deficient CDK9 mutant-encoding plasmid, they tested whether CDK9 kinase activity is important for the TRAF2-mediated activation of NF-κB induced by TNFα. However, the results from these experiments were inconsistent: Both the expression of the CDK9 mutant and of wild-type CDK9 exhibited a decreased NF-κB activity. The authors hypothesized that the overexpression of the wild-type CDK9 causes an increased nuclear localization, which does not allow to detect cytosolic effects. In 2002, De Falco *et al.* [88] confirmed that CDK9 can also be present in the cytoplasm. Furthermore, they described that CDK9 associates with gp130 upon IL-6 stimulation. The transmembrane glycoprotein gp130 is an important signal transducer and subunit of each of the receptors that bind members of the IL-6 cytokine family. It is noteworthy that, despite this knowledge about the binding of CDK9 to TRAF2 and gp130, it remains unclear whether anti-inflammatory effects of CDK9 inhibitors are linked to these two pro-inflammatory signal transducers.

## CONCLUSION

Targeting CDK9 with small molecule inhibitors represents a viable strategy for the treatment of several diseases, indicated especially by the deregulation of CDK9 activity in cancers, cardiac hypertrophy, HIV infections and pathological inflammation. However, gaps in our knowledge of CDK9 and its inhibition to date have meant that a therapeutic application remains a long way off: (i) Unfortunately, many CDK9 inhibitors are unselective, targeting several CDKs and other kinases, thus causing significant off-target associated toxicity. Inhibitors with a better selectivity for CDK9 need to be developed. The available CDK9 X-ray structres can guide the design of such inhibitors. (ii) CDK9 inhibition (solely by flavopiridol) has as yet only been tested in two inflammatory *in vivo* models (arthritis, hepatitis). Other pathologically relevant disease models have to be investigated. (iii) It is of the utmost importance to confirm that CDK9 is indeed the critical target for the induction of anti-inflammatory effects by CDK9 inhibitors *in vivo*. (iv) The role of CDK9 in the respective disease-relevant cell types has to be revealed on a cellular-functional and molecular-mechanistic basis in much more detail in order to precisely understand its mode of action. 

## Figures and Tables

**Fig. (3):**
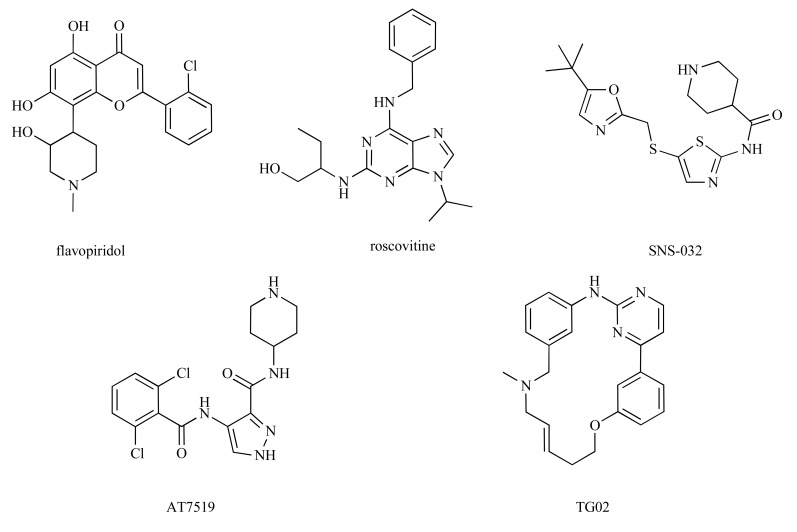
Structures of some clinically evaluated CDK inhibitors discussed in the review.
